# The Medical Society of the State of New York

**Published:** 1901-03

**Authors:** 


A Monthly Review of Medicine and Surgery.

*EDITOR:*

WILLIAM WARREN POTTER, M. D.

All communications, whether of a literary or business nature, books for review and exchanges should be addressed to the editor: 284 FRANKLIN STREET, BUFFALO, N. Y.

VOL. XL.—LVI. MARCH, 1901. No. 8.

### THE MEDICAL SOCIETY OF THE STATE OF NEW YORK.

THE ninety-fifth annual meeting of the Medical Society of the State of New York was held at Albany, January 29, 30 and 31, 1901. From all sides the verdict rendered is, that from a scientific, social and economic standpoint, it took rank as one of the better meetings in the history of the society. The presence and coöperation of the President of the American Medical Association, Dr. Charles A. L. Reed, of Cincinnati, O., added not a little to the interest and good feeling manifested by the large number of representative physicians present from all parts of the Empire State.

The President of the Society, Dr. A. M. Phelps, of New York, directed affairs with forceful dignity and with the aid of the business committee, under the chairmanship of Dr. Frank Van Fleet, of New York, despatched an enormous amount of business during the short space of time at the disposal of the society. Perhaps it might be as well in the future to curtail the number of scientific communications, and give each contributor sufficient time to finish his contribution and allow reasonably untrammeled discussion.

The president in his inaugural address advocated a number of innovations, prominent among which were the organisation of an Atlantic Coast Medical Association, similar to the interstate associations in the west, an amendment to the constitution allowing each county society to send two delegates annually from each assembly district, and the holding of a semi-annual scientific session of the society in the fall of the year in one of the large cities of the state. His remarks were given close attention.

The report of the standing committees were as usual of the utmost interest and importance; especially so to the Buffalo members were the ones on hygiene and prize essays. Dr. Henry R. Hopkins's report, as chairman of the committee on hygiene, printed in full in this journal, was a masterful review of the sanitary conditions existing, and the progress making in the housing of the poor, the water supplies of cities, and care of the incipient cases of tuberculosis. The prize essay committee, of which Dr. Abraham Jacobi, of New York, is chairman, announced the award of the Merritt H. Cash prize for 1901, to Dr. Lucien Howe, of Buffalo.

The president introduced Dr. Charles A. L. Reed, who was enthusiastically received, and spoke as follows:

*Mr. President and Fellows of the Medical Society of the State of New York:* I call you "fellows" in a spirit of fellowship, because I have been one of your fellows, much to my gratification, for the last ten years. It affords me extreme pleasure to stand before you today in response to the cordial invitation of your president. When it was my privilege to appear for the first time before this distinguished body, ten years ago this day, I came simply in my capacity as a humble worker in the ranks of the profession; today I am introduced to you as the president of the national organisation, although no less a humble worker. The terms in which I have been presented to you makes the duty a pleasing one of extending to you, in the most cordial spirit, the greetings of the American Medical Association. I have been informed that such messages have been a trifle unusual in the past (laughter,) and I almost feel that I am in a foreign country, yet it seems to me that you are wrestling with precisely the same problems which have confronted the medical profession in Ohio, and in most of the other states. For a proper solution of this it has been found necessary to have the absolute unity and coöperation of the entire profession. If the American Medical Association stands for one idea over and above another, it is for the unity and for the solidarity of our great national profession. If there is today one spirit actuating it over and above another, it is the spirit which I have expressed in the words just pronounced. This is the one assurance which I bring you, and I am satisfied, from the expressions which I have heard in Albany, that that sentiment is very cordially reciprocated.

I am sure, when we gather in our national council in St. Paul next June, we shall be nearer an understanding on the unfortunate questions which have divided us for nearly two decades, than we have been these many years.

I am gratified, indeed, to notice the expressions which have cropped out in these proceedings this morning, to which I have been a very attentive listener, as they indicate that there is a disposition in your body to be democratic, and to bring within the fold of this society the workers in the ranks of the profession. In my various goings about the country, I have found that those societies seem to be doing the most good and accomplishing the greatest results, that have been able to bring within their ranks the largest numbers of the medical profession. The weak man needs the society, and the strong man is needed by the society; hence we need them all, but the society must be given a proper degree of respectability. Therefore I view with satisfaction the initial step which you have taken today, looking to the broadening of your influence within the limits of your own state, feeling sure that that influence and that solidarity will sooner or later inure to the benefit of the great national body of the profession. I thank you, Mr. President, for your cordial reception." (Prolonged and hearty applause.)

The annual address of the president on the second day on the Radical Cure of Inguinal Hernia, was a comprehensive review of the subject from the commencement of the Christian era down to the beginning of this twentieth century, and the reader asserted that we had arrived at the last stage of perfection in the technique of the operation for its radical cure.

The annual dinner given at the Ten Eyck was attended by a large number of members, delegates and guests.

The officers elected for 1901 are as follows: President, Henry L. Elsner, of Syracuse; vice-president, Louis N. Lanehart, of Hempstead; secretary, F. C. Curtis, of Albany; treasurer, O. D. Ball, of Albany.

It was decided to hold the first semi-annual session in New York City.

